# LncRNA GAS5-hnRNPK axis inhibited ovarian cancer progression via inhibition of AKT signaling in ovarian cancer cells

**DOI:** 10.1007/s12672-023-00764-6

**Published:** 2023-08-28

**Authors:** Te Zhang, Yahui Leng, Mengjing Duan, Zihang Li, Yongqing Ma, Chengyang Huang, Qin Shi, Yi Wang, Chengcheng Wang, Dandan Liu, Xuan Zhao, Shuang Cheng, Ao Liu, Yang Zhou, Jiaqi Liu, Zhongqiu Pan, Huimei Zhang, Li Shen, Hongyan Zhao

**Affiliations:** 1 Hubei Key Laboratory of Embryonic Stem Cell Research, Hubei University of Medicine, Shiyan, Hubei China; 2 Department of Obstetrics and Gynecology, The Third Affiliated Hospital of Chongqing Medical University, Chongqing, China; 3 Biomedical Research Institute, Hubei University of Medicine, 442000 Shiyan, Hubei China; 4 Department of Clinical Oncology, Taihe Hospital, Hubei University of Medicine, 30 South Renmin Road, 442000 Shiyan, Hubei China; 5 Hengdian Central Health Center, Huangpi District, Wuhan, Hubei China; 6 The Second Clinical College, Xi’an Medical University, Xi’an, Shaanxi China

**Keywords:** Ovarian cancer, LncRNA, GAS5, hnRNPK

## Abstract

**Background:**

The incidence of ovarian cancer ranks third among gynecologic malignancies, but the mortality rate ranks first.

**Methods:**

The expression of GAS5 is low in ovarian cancer and is associated with the low survival of ovarian cancer patients according to public ovarian cancer databases. GAS5 overexpression inhibited ovarian malignancy by affecting the proliferation and migratory abilities in OVCAR3 and A2780 cells. GAS5 overexpression increased the rate of cell apoptosis, and the cells were blocked in the G1 phase as assessed by flow cytometry.

**Results:**

We found that hnRNPK was a potential target gene, which was regulated negatively by GAS5 based on RNA-pulldown and mass spectrometry analysis. Mechanistically, GAS5 affected the inhibition of the PI3K/AKT/mTOR pathways and bound the protein of hnRNPK, which influenced hnRNPK stability. Furthermore, rescue assays demonstrated hnRNPK was significantly involved in the progression of ovarian cancer.

**Conclusions:**

Our study showed one of the mechanisms that GAS5 inhibited ovarian cancer metastasis by down-regulating hnRNPK expression, and GAS5 can be used to predict the prognosis of ovarian cancer patients.

**Graphical Abstract:**

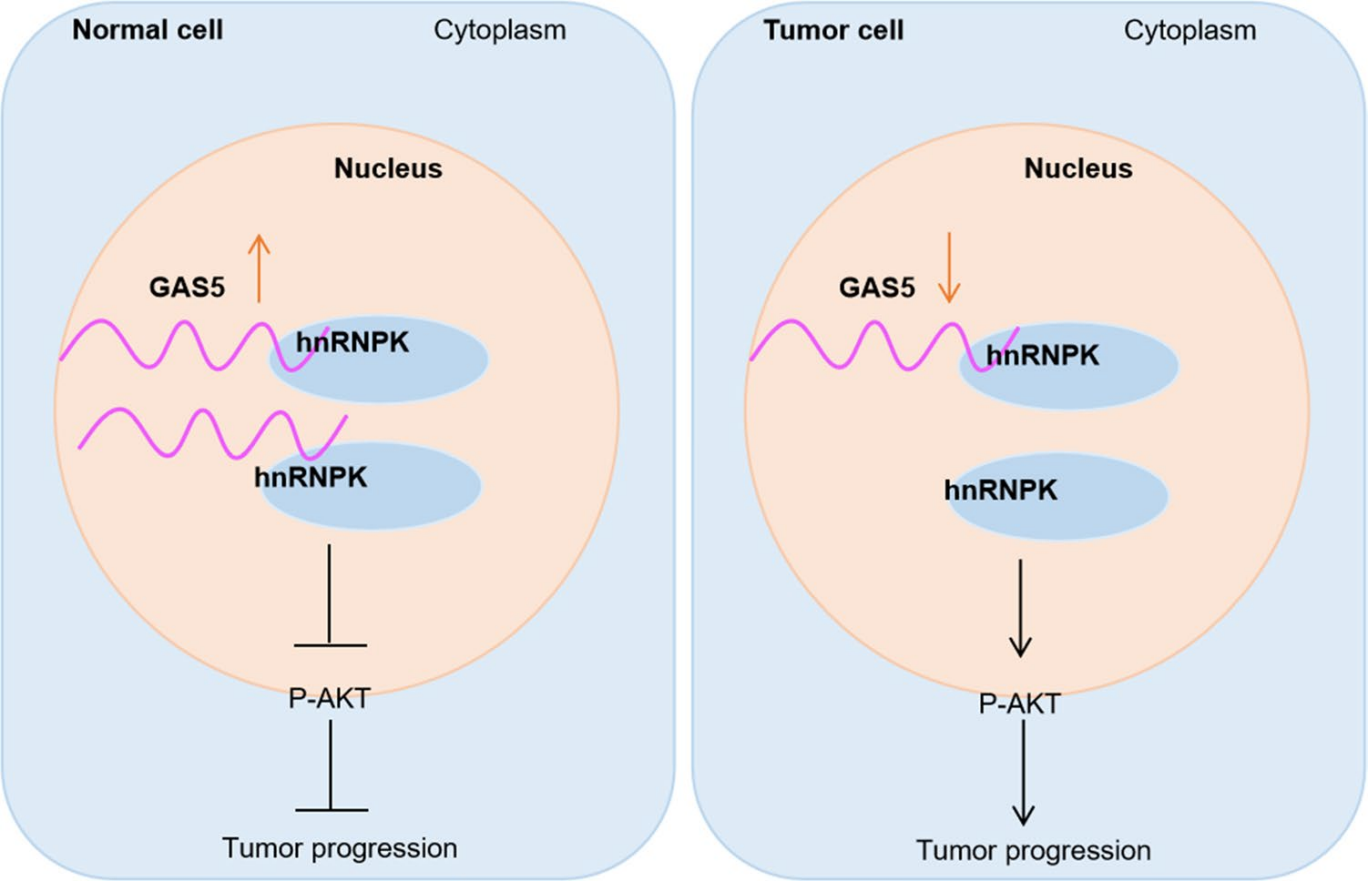

**Supplementary Information:**

The online version contains supplementary material available at 10.1007/s12672-023-00764-6.

## Introduction

The incidence of ovarian cancer ranks third in gynecological malignancy, but the mortality rate ranks first in gynecological cancer, which is the major disease that seriously threatens women's health. Due to the lack of symptoms in the early stage of ovarian cancer, even if there are symptoms, symptoms are not obvious. The method of screening is limited, so early diagnosis is more difficult. 60–70% of patients have been at an advanced stage at the time of treatment. Surgery and chemotherapy are the main means of treatment of ovarian malignant tumors, and there are not effective in advanced stage cases [1–3]. Therefore, it is very important to reveal the molecular mechanism, which will develop new biomarkers or therapeutic targets against ovarian cancer.

Long non-coding RNAs (LncRNAs) are a class of RNA molecules that are transcripts more than 200 nt in length. LncRNAs are generally considered to not encode proteins and are involved in protein-coding gene regulation in the form of RNA at multiple aspects [4]. LncRNAs interfere expression of the adjacent gene, mediate chromatin remodeling and histone modification, affect microRNA binding of the target genes, regulate the function of target genes, interfere with the shearing of protein-coding genes mRNA through complementary with protein-coding genes transcripts, regulate the activity and the cellular localization of proteins by binding specific proteins [5–7]. LncRNAs are precursors molecules of microRNAs and other small RNA molecules by complementation with transcription of protein-coding genes under the action of a dicer enzyme [5–7]. As a structural component, LncRNAs are conducive to forming RNA protein complexes involved in gene expression regulation [8]. LncRNAs have been discovered to play an important role in tumor development. Many LncRNAs are potential biomarkers and targets for the diagnosis and treatment of cancers [9, 10].

LncRNA growth arrest-specific transcript 5 (GAS5), locates chromosome at 1q25 [11]. It was first discovered in mouse NIH3T3 fibroblasts due to increased expression after cell growth arrest [12]. In a variety of tumors, the expression of GAS5 was significantly decreased, suggesting that GAS5 might be a tumor-suppressing LncRNA [13]. GAS5 negatively regulated the expression of microRNA downstream target genes by binding specific microRNA in multiple tumors [14]. Studies reported that GAS5 affected the stability of the P53 protein and inhibited the migration and invasion of gastric cancer by interacting with the P53 protein [15]. However, whether GAS5 affects the occurrence and development of ovarian cancer through interaction with protein remains unclear.

Heterogeneous nuclear ribonucleoprotein K (hnRNPK) belongs to the hnRNP protein family and interacts with nucleic acids and proteins [16]. As a multifunctional protein, hnRNPK participates in transcriptional regulation, translation regulation, RNA cleavage processing, DNA repair, chromatin remodeling and other biological processes [17]. Studies reported that LncRNA CRLM1 promoted hnRNPK nuclear localization by interacting with hnRNPK and affected the occurrence and progression of tumors [18]. Subsequently, assays in vitro were carried out to clarify the biological function of GAS5 and hnRNPK. Mechanistically, GAS5 regulated the expression of hnRNPK and influenced hnRNPK protein stability. Our research is expected to provide a novel therapeutic target for ovarian cancer.

## Materials and methods

### Cell lines and cell culture methods

Ovarian cancer cell lines OVCAR3, A2780, and human embryonic kidney cells HEK293T were purchased from the Cell Center of the Chinese Academy of Medical Sciences (Beijing, China). All cell lines were stored in liquid nitrogen. Cells were regularly examined for mycoplasma contamination. Both OVCAR3 and 293 T were cultured in DMEM (Gibco, USA) medium with 10% FBS (VivaCell, China) and 1% Penicillin streptomycin (Biosharp, China). A2780 was maintained in RPMI1640 (Gibco, USA) with 10% FBS (VivaCell, China). All the cells were cultured at 37℃ in a 5% carbon dioxide incubator.

### Authentication of cell lines

All the ovarian cancer cell lines and 293 T cells have been authenticated using short tandem repeat profiling within the last two years.

### Plasmids

The knockout plasmid used in lentivirus-mediated interference, complementary sense and antisense oligonucleotides were designed, synthesized, annealed, and finally cloned into pLKO.1 vector (Additional file 1: Table S1). GAS5 expression plasmid was cloned into the pcDNA(-) vector (GENEWIZ Biotech, China). The expression plasmid hnRNPK was cloned into a pENTER vector (Vigene Biosciences, China).

### Transfection and infection

Transfection: GAS5 and hnRNPK expression plasmids were transiently transfected into ovarian cancer cells by using TransIT-LT1 Transfection (Mirus, USA) when the confluence of the cells was 80%. The cells were collected and the transfection efficiency was checked by RT-qPCR or Western blotting assays after 48 h of transfection.

Infection: hnRNPK knockdown plasmids were termed as shPK. Control lentivirus was termed NC. NC and shRNA vectors were transfected into HEK293T cells with packaging vectors psPAX2 (#12260, Addgene) and pMD2.G (#12259, Addgene) using TransIT-LT1 Transfection (Mirus, USA). Lentivirus particles were harvested at 60 h after transfection, and filtered through 0.22 μm PVDF filters. When the confluence of the cells was 30%, we performed lentivirus infection.

### RNA isolation and RT-qPCR

Total RNA was extracted from A2780 and OVCAR3 cells using Trizol reagent (Invitrogen, USA). The qPCR primers were designed by the primer bank website and synthesized at the company (TSINGKE, China) (Additional file 1: Table S1). The amount of RNA used for the cDNA synthesis reaction was 2 μg. Reverse transcription and quantitative real-time polymerase chain reaction (qRT-PCR) were performed using kits (Vazyme, China) on Bio-rad CFX96 real-time PCR system (Biorad, USA) to detect the RNA expression levels. GAPDH or Actin was used as the internal standard control for qRT-PCR. Target gene levels were normalized to control RNA levels, and relative gene expression was calculated using the 2^−△△CT^ method.

### Western blotting and antibodies

Total protein concentration was determined using BCA Protein Assay Kit (Solarbio, China). The protein was separated by 10% SDS-PAGE and then transferred onto 0.22 μm PVDF membrane (Millipore, USA). All the protein-blotted PVDF membrane was blocked in 5% non-fat milk in TBST and incubated with specific antibodies. Antibodies were used as follows: hnRNPK (ab70492, Abcam, UK), anti-GAPDH (6004-1-1 g, Proteintech, China), anti-AKT (abs131789, Absin, China), anti-P-AKT (abs130002, Absin, China), anti-PI3K (abs131198, Absin, China), anti-P-PI3K (abs130868, Absin, China), anti-mTOR (abs131824, Absin, China), anti-P-mTOR (abs130935, Absin, China), anti-mouse secondary antibodies, anti-rabbit secondary antibodies (Proteintech, China). Protein levels were detected using an Immobilon Western Chemilum HRP Substrate (Millipore, USA). The expression of the protein was analyzed by grayscale using Image-Pro Plus 6.0 software.

### Cell growth and proliferation assays

Cells were collected and then counted with Countstar (Countstar Automated Cell Counter, China). For growth assay, 2000 or 2500 cells were inoculated into 96-well plates and cultured at 37 ℃ in a 5% carbon dioxide incubator for 0 h, 24 h, 48 h, 72 h, and 96 h. Incubated plates for another 2 h in the incubator after adding 10% CCK8 (DOJINDO, Japan) into each well, and the 450 nm absorbance was measured as cell viability.

For the proliferation assay, 2000 or 3000 cells were inoculated into 6-well plates and cultured for about 10 days at 37 ℃ in a 5% carbon dioxide incubator. Cells were fixed with 4% paraformaldehyde for 30 min and stained with crystal violet (Sigma-Aldrich, USA) for 1 h. After being washed twice with PBS, the number of cell clones was counted using ImageJ software.

### Transwell migration and invasion assays

Cells were collected and counted with Countstar (Countstar Automated Cell Counter, China). Migration assays were performed using a 24-well Transwell chamber system (Millipore, USA). 1 × 10^5^ cells were resuspended with 0.2 ml serum-free culture medium and seeded in the upper chamber. The lower chamber added 0.6 ml culture medium with 20% serum. For invasion assays, the upper chamber was coated with Matrigel (Corning, USA) before plating cells. After incubating at 37 °C carbon dioxide incubator for 24 h, the cells were fixed with 4% paraformaldehyde for 30 min, and stained with crystal violet (Sigma, USA) for 1 h. Washed the stained cells using PBS, and wiped off non-invasive cells with a cotton swab in the upper chamber. Migrated or invaded cells were imaged under a 20 × microscope and counted using ImageJ software.

### Apoptosis and cell cycle detection

The cells were harvested and detected apoptosis levels by Annexin V-FITC apoptosis detection kit (Beyotime, China) according to the instructions of the procedure. The apoptosis was analyzed by flow cytometry. The apoptosis rate was examined with the Annexin V + PI + cells method. For cell cycle analysis, the cells were fixed with 70% ethanol overnight at 4 °C and then the cells were incubated with propidium iodide (PI) (Beyotime, China) and RNase (TIANGEN China), the percentage of cells in G1, G2, and S phases was analyzed by CytExpert and Modfit software. Each experiment was conducted in 3 biological replicates.

### RNA pull-down and mass spectrometry

Biotin-labeled GAS5 were transcribed in vitro with the Biotin RNA Labeling Mix and T7 RNA polymerase (Thermo Fisher Scientific, USA), and then incubated with biotin-labeled GAS5 probe and Streptavidin Magnetic Beads (Thermo Fisher Scientific, USA) at 4 °C overnight. Total cell lysates were added to each reaction and then incubated on a rotor for 4 h at 4 °C. After washing three times, the RNA–protein binding mixture was boiled in SDS buffer. Finally, the retrieved protein was processed for Coomassie Brilliant Blue staining and send to mass spectrometry analysis (PTM BIO, China) (Additional file 2: Table S2).

### RNA immunoprecipitation

Approximately 1 × 10^7^ OVCAR3 and A2780 cells were harvested, washed with PBS, and resuspended in 0.5 mL IP lysis buffer (20 mM HEPES, 150 mM NaCl, 0.5 mM EDTA, 10 mM KCl, 0.5% NP-40, 10% glycerol, 1.5 mM DTT, 1 mM PMSF, RNase inhibitor [10 U/mL]). The cells were lysed on ice for 30 min and then centrifuged at 12000 rpm for 15 min. The cell lysates were collected and incubated with anti-hnRNPK (4 μg) or control IgG (4 μg) antibody and 30 μL of Protein G beads (Invitrogen, USA) at 4 °C overnight. The mixtures were washed three times with lysis buffer, the 1 mL Trizol Reagent was added to the mixtures and the RNA was extracted for qRT-PCR.

### Protein stability assay

To test protein stability, OVCAR3 and A2780 ovarian cancer cells were transfected with pcDNA3.1 and GAS5 plasmids. The transfected cells were seeded into a 6-well plate and exposed to 2 mg/mL cycloheximide (MCE, USA). The cells were incubated at 37 °C for 0, 3, 6, 9, and 12 h. Total protein was extracted for Western Blotting detection.

### Statistical analysis

Statistical analysis was performed using GraphPad Prism 9. Quantitative results were expressed as the mean ± standard error of the mean (SEM) and were analyzed using multiple t-tests. P-values < 0.05 were considered statistically significant.

## Results

### GAS5 is lowly expressed in ovarian cancer and a poor prognostic factor in a variety of tumors

**Fig. 1:**
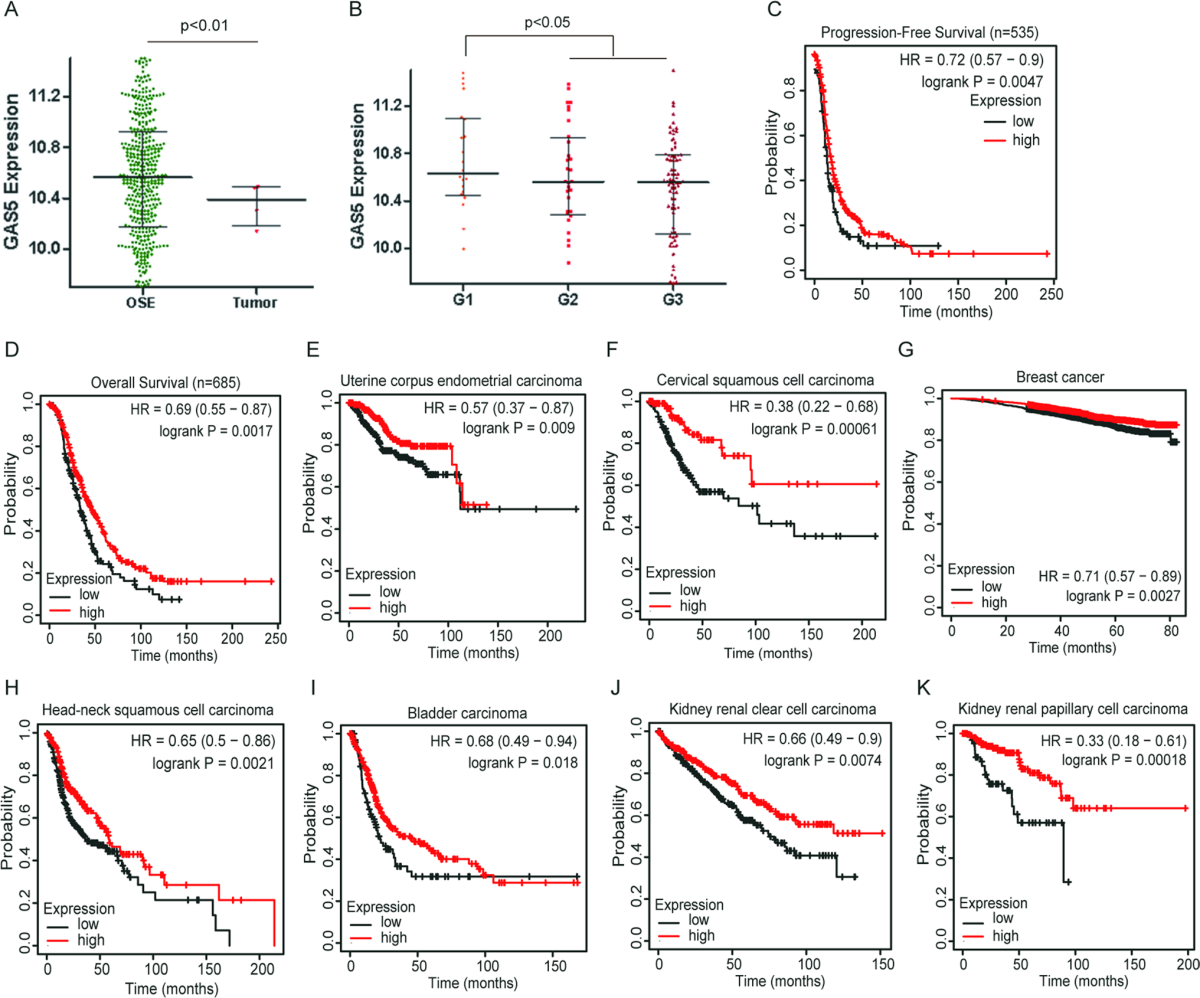
Low expression of GAS5 was associated with tumorigenesis and poor survival of cancer patients. **A** The expression of GAS5 in ovarian cancer was analyzed according to the CSIOVDB database. **B** The relationship between the expression of GAS5 and tumor pathological grade was analyzed by the CSIOVDB database. **C**, **D** Progression-free survival (PFS) and overall survival (OS) curves of OC patients with low or high GAS5 expression by Kaplan–Meier analysis. **E–K** Overall survival (OS) analysis of different cancer patients with low or high GAS5 expression by Kaplan–Meier analysis. Data are shown as mean ± S.D. *P < 0.05, **P < 0.01, ***P < 0.001

We first analyzed GAS5 expression in ovarian cancer according to the CSIOVDB database and found that GAS5 was downregulated in ovarian cancer compared with that in ovarian surface epithelial (Fig. 1A). GAS5 expression in serous ovarian cancer was significantly correlated with tumor pathological grade (Fig. 1B). Additionally, low GAS5 expression was significantly associated with poor overall survival (OS) and progression-free survival (PFS) in ovarian cancer patients through Kaplan–Meier analyses (Fig. 1C, D). To determine the prognostic values of GAS5 in pan-cancer, Kaplan–Meier survival analysis was performed. We found that low GAS5 expression is closely associated with poor prognosis in a variety of tumors (Fig. 1E–K).

### GAS5 affected the proliferation and metastasis of ovarian cancer cells

**Fig. 2:**
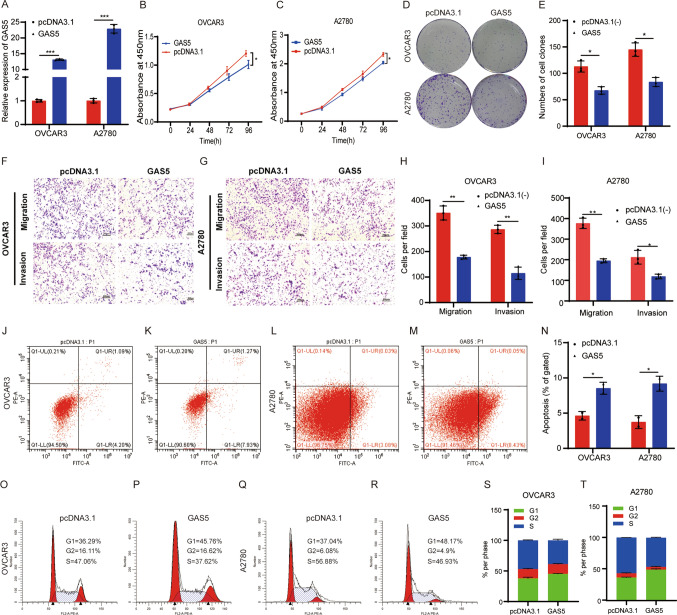
GAS5 inhibited cell proliferation, migration, and invasion and promoted cell apoptosis. **A** Detection of GAS5 expression by RT-qPCR. **B**, **C** Cell proliferation assay. **D** Colony formation assay. **E** Statistical results of colony formation assays. **F**, **G** Transwell migration and matrigel invasion assays (magnification, × 100) Scale bar, 100 μm. **H**, **I** Statistical results of transwell migration and matrigel invasion assays. **J–M** Analysis of cell apoptosis by flow cytometry. **N** Statistical results of cell apoptosis. **O–R** Flow cytometric analysis of cell cycle. **S**, **T** Statistical results of cell cycle assays. All assays were biologically replicated three times. Data are shown as mean ± S.D. *P < 0.05, **P < 0.01, ***P < 0.001

To explore the functions of GAS5 in ovarian cancer, we characterized the cellular phenotypes of ovarian cancer cells with overexpression of GAS5. We investigated the effects of overexpressing GAS5 on the growth and metastasis of ovarian cancer cell lines including OVCAR3 and A2780 (Fig. 2A). GAS5 overexpression inhibited the growth of ovarian cancer cell lines as determined by (Cell Counting Kit-8) CCK-8 and colony formation assays (Fig. 2B–E). Transwell assays demonstrated that overexpressing GAS5 inhibited the migration and invasion of ovarian cancer cells (Fig. 2F–I). To further determine the cell cycle progression and apoptosis effect of GAS5 in OVCAR3 and A2780 cells, we conducted flow cytometry to detect the cell-cycle distribution and cell death rate. Overexpression of GAS5 increased the death rate of ovarian cancer cells by comparing early apoptosis (Q1-LR) values using flow cytometry (Fig. 2J–N). Besides, overexpression of GAS5 increased the accumulation of ovarian cancer cells in the G1 phase (Fig. 2O–T). These data illustrated that GAS5 exerted tumor-inhibiting properties in ovarian cancer.

### hnRNPK is a target of GAS5 in ovarian cancer

**Fig. 3:**
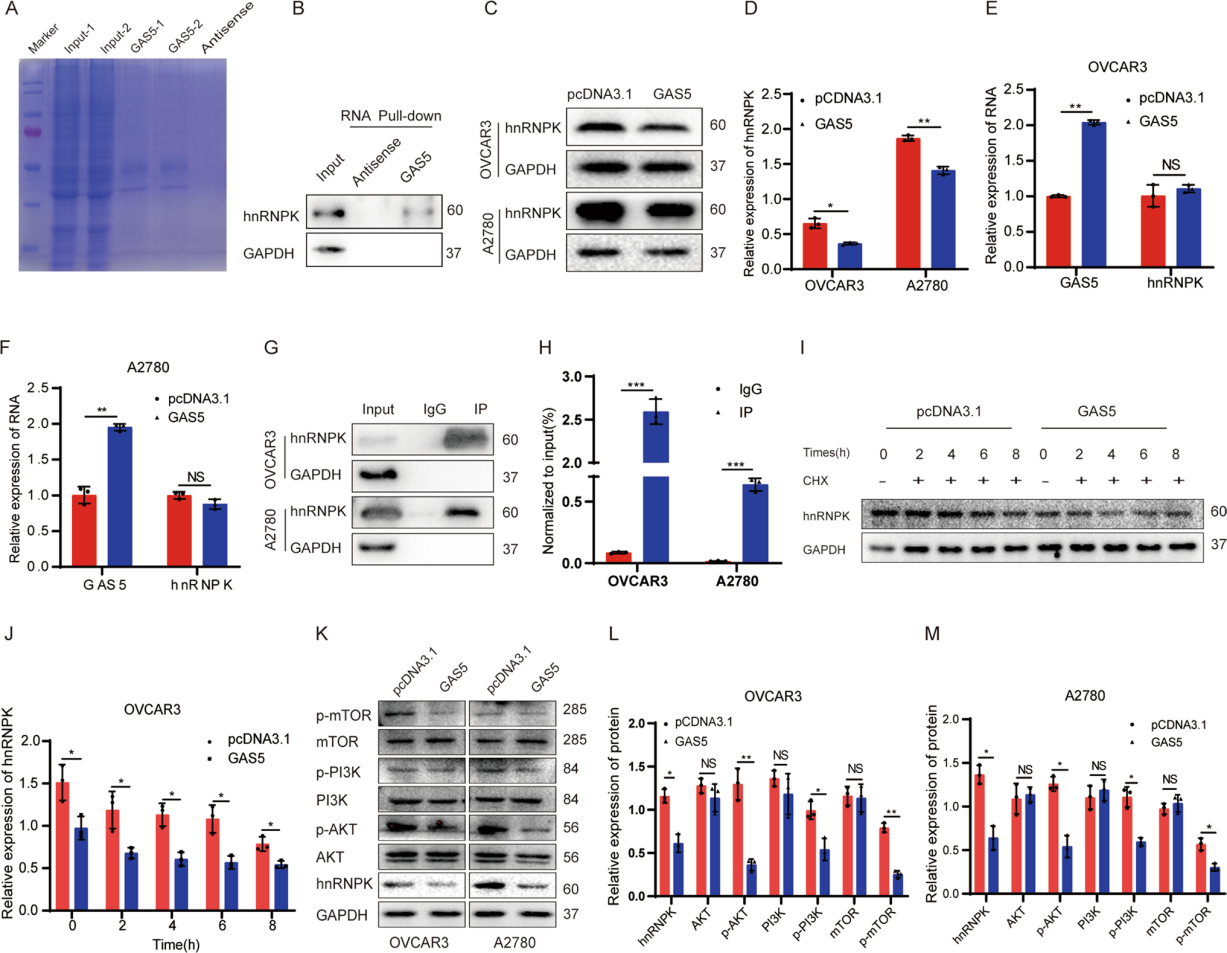
GAS5 regulated hnRNPK protein stability in ovarian cancer cells. **A** Coomassie Brilliant Blue Staining after GAS5 pull down. **B** Western blotting assay verifying the enrichment of hnRNPK in RNA-pulldown assays. **C** Detection of hnRNPK expression by western blotting following GAS5 overexpression. **D** The statistics results of hnRNPK protein by grayscale in (**C**). **E**, **F** Detection of GAS5 and hnRNPK expression by RT-qPCR. **G** Western blotting verification RIP assay effective. **H** RT-qPCR assay verifying the enrichment of GAS5 in RIP assays. **I** The protein stability assay. **J** The statistics results of stability assay by grayscale in (**I**). **K** Effects of GAS5 overexpression on the expression of PI3K/AKT/mTOR signaling pathway.** L**, **M** The expression of the protein was quantified by grayscale in (**K**). All assays were biologically replicated three times. Data are shown as mean ± S.D. *P < 0.05, **P < 0.01, ***P < 0.001. NS, not significant

To reveal the regulatory mechanism of GAS5 in ovarian cancer, we used RNA pull-down and mass spectrometry analysis to screen the proteins co-interacting with GAS5. Coomassie Brilliant Blue Staining suggested RNA pull-down effectively (Fig. 3A, Additional file 2: Table S2). The 5% input was taken and western blotting indicated that hnRNPK was enriched using RNA pull-down assay (Fig. 3B). When ovarian cancer cells were transfected with the GAS5 plasmids, Western blotting verified that hnRNPK protein was downregulated (Fig. 3C, D) and the RNA expression of hnRNPK was no significant change by RT-qPCR (Fig. 3E, F). To explore the interaction between hnRNPK and GAS5, we performed hnRNPK RIP assays (Fig. 3G). The RT-qPCR showed that GAS5 was efficiently enriched by hnRNPK RIP assays (Fig. 3H). To evaluate whether GAS5 impacted the stability of hnRNPK, the protein stability assay was performed. Interestingly, GAS5 affected the stability of the hnRNPK protein (Fig. 3I, J). To validate the association between PI3K/AKT/mTOR signaling pathways and GAS5, the total protein and phosphorylation levels of PI3K(Tyr607), AKT(Ser473), and mTOR(Thr2446) were examined via Western blotting. As expected, the phosphorylation levels of PI3K, AKT, and mTOR, but not the total levels of PI3K, AKT and mTOR, were reduced following GAS5 overexpression (Fig. 3K–M). The research verified that overexpression of GAS5 inhibited the PI3K/AKT/mTOR signaling pathway in ovarian cancer cells.

### Oncogenic role of hnRNPK in ovarian cancer cells and a poor prognostic factor in a variety of tumors

**Fig. 4:**
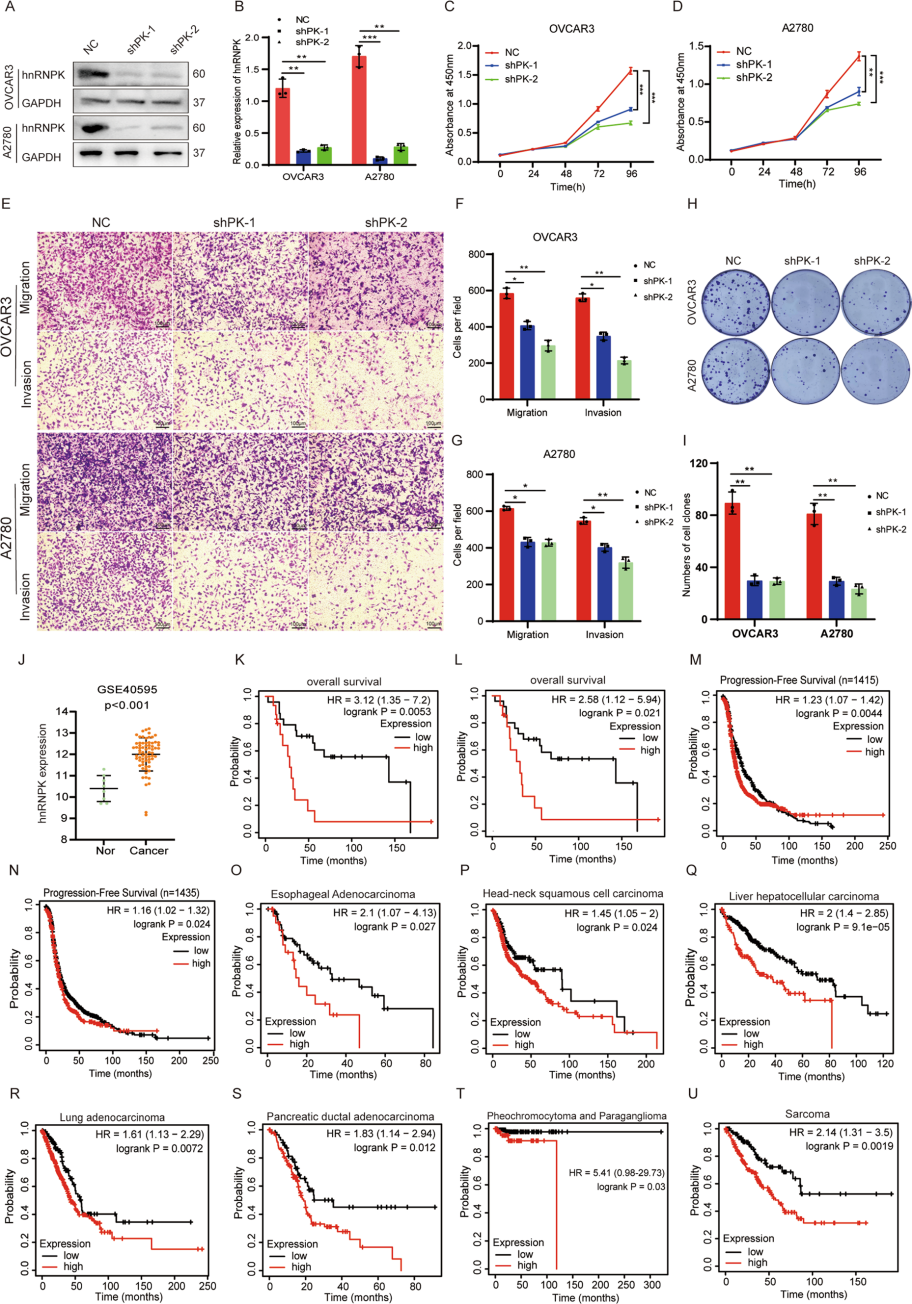
hnRNPK promoted proliferation and metastasis of ovarian cancer cells and promoted tumorigenesis. **A** Detection of hnRNPK expression by Western blotting. **B** The statistics results of hnRNPK protein by grayscale in (A). **C**, **D** Cell proliferation assay. **E** The migration and matrigel invasion assays (magnification, × 100) Scale bar, 100 μm. **F**, **G** The statistical results of transwell migration and matrigel invasion assays in (E). **H** The colony formation assays. **I** The statistical result of colony formation assays in (H). **J** Analysis of hnRNPK protein expression in ovarian cancer according to GEO datasets. **K–M** Progression-free survival (PFS) and overall survival (OS) curves of OC patients with low or high hnRNPK expression by Kaplan–Meier analysis. **N–U** Overall survival analysis of cancer patients with different hnRNPK expression by Kaplan–Meier analysis. All assays were biologically replicated three times. Data are shown as mean ± S.D. *P < 0.05, **P < 0.01, ***P < 0.001

To elucidate the biological function of hnRNPK in ovarian cancer cells, hnRNPK was knocked down in OVCAR3 and A2780 cells using shRNAs. Successful knockdown of hnRNPK in OVCAR3 and A2780 cells was confirmed by Western blotting (Fig. 4A, B). The hnRNPK deficiency repressed ovarian cancer cell growth as determined by CCK-8 assays (Fig. 4C, D) and inhibited the colony formation abilities of ovarian cancer cells (Fig. 4H, I). Transwell assays demonstrated that hnRNPK deficiency inhibited the migratory and invasive abilities of ovarian cancer cells (Fig. 4E–G). Together, these functional studies illustrated that hnRNPK exerted a tumor-promoting property in ovarian cancer cells. Meanwhile, we analyzed hnRNPK expression in ovarian cancer using the GEO database. The results showed that hnRNPK was significantly upregulated in ovarian cancer compared with normal ovarian tissues (Fig. 4J). High hnRNPK expression was significantly associated with poor overall survival (OS) and progression-free survival (PFS) of ovarian cancer patients (Fig. 4K–N). Finally, we evaluated hnRNPK prognostic values in pan-cancer (Fig. 4O–U). The results revealed that hnRNPK was significantly increased and closely associated with poor OS in a variety of tumors.

### Overexpression of hnRNPK ameliorated the tumor-suppressive effect of GAS5 in ovarian cancer cells

**Fig. 5:**
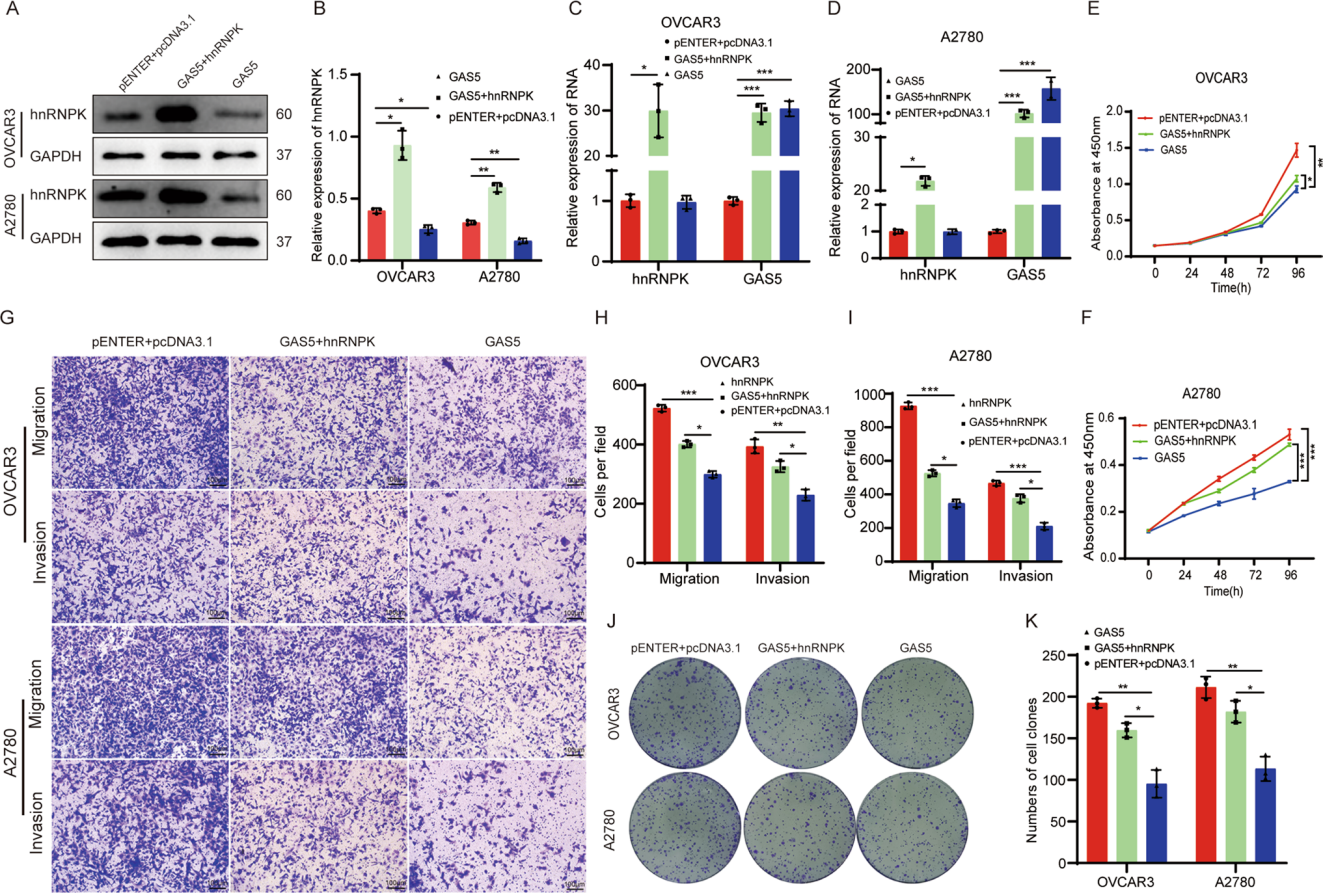
GAS5 inhibited cell proliferation and migration of ovarian cancer cells dependent on hnRNPK. **A** Detection of hnRNPK expression by Western blotting in GAS5 overexpression ovarian cancer cells with overexpression of hnRNPK. **B** The expression of hnRNPK protein was analyzed by grayscale in (**A**). **C**, **D** Detection of GAS5 and hnRNPK expression by RT-qPCR. **E**, **F** Cell proliferation assay. **G** The migration and matrigel invasion assays (magnification, × 100) Scale bar, 100 μm. **H**, **I**. The statistical results of transwell assays in (**G**). **J** The colony formation assays. **K** The statistical result of colony formation assays in (**J**). All assays were biologically replicated three times. Data are shown as mean ± S.D. *P < 0.05, **P < 0.01, ***P < 0.001

To further verified the association of hnRNPK with the tumor-suppressive effect role of GAS5 in ovarian cancer, hnRNPK was overexpressed in GAS5 overexpression ovarian cancer cells. Western blotting showed that the amount of hnRNPK protein was increased in all the GAS5 overexpression cells after hnRNPK overexpression (Fig. 5A, B). RT-qPCR showed the RNA expression of hnRNPK was unchanged (Fig. 5C, D). GAS5 inhibited cell growth and proliferation, whereas overexpression hnRNPK reversed this phenomenon by CCK-8 and colony formation assays. (Fig. 5E, F, J and K). The suppressive effect of GAS5 on migration and invasion in ovarian cancer cells was reversed by overexpression of hnRNPK by transwell assays (Fig. 5G–I). These results indicate that hnRNPK may be a key downstream target of GAS5 that inhibits ovarian cancer progression.

## Discussion

Our results revealed that GAS5 showed a tumor suppressor function in ovarian cancer. In terms of mechanism, the research results showed that GAS5 affected the protein stability of hnRNPK, and then affected the occurrence and development of ovarian cancer.

LncRNAs are generally considered to not encode proteins and are involved in protein-coding gene regulation in the form of RNA at multiple levels including epigenetic regulation, transcriptional regulation, and post-transcriptional regulation [19]. The function of most lncRNAs is closely associated with RNA-binding proteins (RBPs) [19]. LncRNAs regulate protein activity and change protein localization by binding to specific proteins. Studies have reported that LncRNA EPIC1 promotes cell cycle progression by interacting with MYC through EPIC1’s 129–283 nt region in Cancer [20]. LncRNA GUARDIN affects genomic stability by binding P53 protein [21]. GAS5 interacts with the WW domain of YAP to facilitate the translocation of endogenous YAP from the nucleus to the cytoplasm and promotes phosphorylation and subsequently ubiquitin-mediated degradation of YAP [22]. In hepatocellular carcinoma, GAS5 attenuates cell viability and invasion by boosting p21 expression via binding YBX1 [23]. LncRNAs bind to RNA-binding protein (RBP) forming the LncRNA-protein complex, which affects gene expression, protein stability, transport, transcription and localization [24, 25]. The protein of mass spectrometry analysis following RNA pull-down included cell structural protein, a lot of RNA binding proteins and proteins associated with tumor development. The protein of hnRNPK ranks ninth by the score of mass spectrometry analysis.

Heterogeneous nuclear ribonucleoproteins (hnRNPs) are a family of proteins with central roles in processes such as splicing control, mRNA stability, cancer-immune, telomere biogenesis, cytoplasmic trafficking of mRNAs, and many hnRNP proteins participates in tumor development [26–28]. hnRNPA2 regulates alternative mRNA splicing of TP53INP2 to control invasive cell migration in ovarian cancer [29]. Small interfering RNA-mediated reduction in heterogeneous nuclear ribonucleoparticule A1/A2 proteins induces apoptosis in multiple cancer cells [30]. Loss of hnRNPA2B1 inhibits malignant capability and promotes apoptosis via down-regulating Lin28B expression in ovarian cancer [31]. The hnRNPK promoted ovarian cancer development by cell function assays. The other hnRNPs proteins were also identified in mass spectrometry including hnRNPKM, hnRNPL, and so on. These results suggest that hnRNPs proteins play an important role in ovarian cancer.

RNA-binding protein (RBP) has a highly dynamic regulation process and important biological functions. RBP controlled the processing and transportation of RNA, including regulating RNA splicing, polyadenylation, mRNA stability, mRNA localization, and translation [32, 33] As an RNA-binding protein, hnRNPK promoted the occurrence and development in many tumors by regulating RNA or protein expression [34–36]. In our research, hnRNPK facilitated the growth and proliferation of ovarian cancer.

The interactions between RNA and proteins, played an important role in the growth and metabolism of organisms. GAS5 affected the stability of p53 protein depending on the exon 12 of GAS5 [15]. We speculated that GAS5 inhibited the stabilization of hnRNPK protein which impacted the expression of hnRNPK and the development of ovarian cancer due to the exon 12 of GAS5.

The PI3K/AKT/mTOR signal pathway is involved in cell growth, proliferation, migration, invasion and cell cycle [37, 38]. The PI3K/AKT/mTOR signal pathway was demonstrated to be activated in ovarian cancer [39]. Our research showed GAS5 overexpression inhibited tumorigenesis and malignant progression which was related to the inhbition of the PI3K/AKT/mTOR signal pathways. According to Kaplan–Meier survival analysis, we find that GAS5 is low expression in a variety of tumors. The results suggest that we can use inhibitors of the PI3K/AKT/mTOR pathway to inhibit ovarian cancer development. Our research provided new insights into the mechanism by which GAS5 inhibited the progression of ovarian cancer.

In summary, the finding of this study illustrates the vital function of GAS5-mediated hnRNPK protein stability in ovarian cancer. Our study enriches the molecular mechanism of GAS5 and regulates ovarian cancer progression via hnRNPK, which provided a novel therapeutic strategy to treat ovarian cancer.

### Supplementary Information

**Additional file 1: Table S1.** The sequences of primer and shRNAs.

**Additional file 2: Table S2.** The results of the mass spectrum.

## Data Availability

The data used to support the findings of this study are available from the corresponding author upon request.
